# Hard X‐ray spectromicroscopy of Ni‐rich cathodes under in situ liquid heating conditions

**DOI:** 10.1111/jmi.13403

**Published:** 2025-03-26

**Authors:** Gea T. van de Kerkhof, Carmen Murphy, Shahul H. Abdulrahman, Timothy Poon, Chris Hawkins, Mengliu Li, Angela E. Goode, Julia E. Parker, Manfred E. Schuster

**Affiliations:** ^1^ Diamond Light Source, Harwell Science and Innovation Campus Didcot UK; ^2^ Johnson Matthey Technology Centre, Johnson Matthey, Sonning Common Reading UK

**Keywords:** hard X‐ray spectromicroscopy, in situ liquid heating, MEMS chips, nanoprobe, nano‐XANES, Ni‐rich cathodes

## Abstract

In situ microscopy involves imaging of samples under real reaction conditions. For electron microscopy, micro‐electromechanical systems (MEMS) chips have previously been developed that can hold a liquid or gas inside the vacuum of the electron microscope, with electrical contacts that allow for heating or biasing of the sample. These chips have paved the way for high‐resolution imaging of dynamic chemical reactions. Here, we report the use of such MEMS chips in an in‐house developed setup for a hard X‐ray nanoprobe, applied to Ni‐rich cathode materials. We investigate the chemical and structural changes in nickel‐rich cathodes upon exposure to electrolyte and under heating conditions using hard X‐ray spectromicroscopy. As such, we find marked differences in the behaviour of pure LiNiO_2_ compared to Co and Mn substituted material, NMC811. The use of hard X‐ray spectromicroscopy allows for imaging and observation of: (i) the oxidation state of nickel, changing from Ni^3+^ to Ni^2+^, (ii) the effect of a preexisting fracture in the sample and (iii) the structural degradation of the sample during accelerated aging.

## LAY DESCRIPTION

To visualise dynamic changes in the chemistry of materials microscopists can use so‐called in situ methods. With in situ microscopy the sample is placed in an enclosure where it is subjected to conditions that reflect the material's operating conditions. For electron microscopy, chips have previously been developed that can hold liquids and contain electrical contacts that allow for heating of the sample or to run an electric current through it. These chips have paved the way for high‐resolution imaging of dynamic chemical reactions inside the electron microscope.

However, in situ liquid electron microscopy can be challenging because the electron beam gets scattered strongly by the liquid. Additionally, while electron microscopy is perfect for looking at things at high resolution, it is less capable of creating a large‐scale overview that gives context to the high‐resolution data. On both counts hard X‐ray spectromicroscopy, which combines spectroscopy with microscopy to create chemical maps of the sample, offers a good complementary technique. Hard X‐rays can go through liquid without losing much signal, and X‐ray nanoprobes (meaning X‐ray microscopes at the nanorange) operate at larger length‐scales than transmission electron microscopes.

A field of research that benefits from such a combination of electron and X‐ray microscopy is batteries. In situ offers a great advantage for such studies, as it reveals the dynamics inside the battery during operation or when exposed to high temperatures during storage. A type of battery material that is of particular interest is the nickel‐rich cathode. These cathodes have an incredibly high capacity in their purest form where nickel content is maximised. However, such cathodes suffer from stability issues. To solve these issues, part of the nickel is sometimes replaced by other metals such as cobalt. This stabilises the battery, but in return it loses some capacity.

Here, we present the application of in situ X‐ray spectromicroscopy to nickel‐rich battery materials. The setup used here uses chips developed for electron microscopy, so that the technique could be used for complementary imaging with both X‐rays and electrons under the same conditions. We show how this setup can be used to find out how two nickel‐rich cathodes, one stabilised with cobalt and the other not, react when subjected to high temperatures. The results can help to improve the design of the cathodes, while showcasing how this setup can be used to acquire important information on the stability of battery materials at high temperatures.

## 1 INTRODUCTION

Studying dynamic interactions in chemical processes can reveal crucial information on the reactions that occur. However, revealing such dynamics can be challenging, especially when working with microscopy techniques that require specific operating conditions. As such, in situ imaging requires the development of customised sample environments that can be used to subject the material to realistic operating conditions. Such in situ environments have been particularly successful in electron microscopy (EM), where micro‐electromechanical systems (MEMS) chips allow for studying liquid or gaseous samples inside the vacuum of the microscope.^1^, ^2^, ^3^ This technique has produced crucial insights into the dynamic chemistry of many different types of materials and reactions, including but not limited to catalysts,^4^ corrosion chemistry^5^ and energy materials.^6^, ^7^ One area that benefits particularly from such in situ studies are batteries, where heating and electric biasing have successfully been applied to study the electrochemistry inside the cell, but also to investigate the stability of electrode materials.^8^

A field within battery research for which in situ imaging is of particular interest concerns the chemical and structural stability of cathode materials that are rich in nickel. Ni‐rich cathodes such as LiNiO_2_ are some of the most promising high‐capacity cathodes for lithium‐ion batteries. However, they typically struggle with underlying stability issues^9^ such as gassing,^10^ resistance growth,^11^ microcracking,^12^ structural collapse,^13^ formation of inactive NiO^14^ and/or metal dissolution.^15^ Many of these issues arise from the high temperatures generated inside the battery during long‐term operation or hot storage. Although some of these problems can be counteracted by stabilising the cathode with other transition metals such as Mn, Co or Al,^16^ the demand for improving the Ni content and decreasing the Co content of the cathode materials warrants the understanding of the underlying chemical reactions in the degradation process.

While bulk X‐ray spectroscopy and diffraction can give valuable insights into these processes,^11^, ^13^, ^14^ they fail to capture localised changes in the cathode structure. For this, EM can provide a valuable tool,^17^, ^18^ but while transmission electron microscopy (TEM) can provide valuable high‐resolution images it lacks the larger overview needed to place the images in a wider spatial context. Additionally, liquid in situ EM suffers from strong electron scattering by the liquid that complicates spectroscopy and high‐resolution imaging.^19^, ^20^ Ex situ EM does not suffer from these limitations but can only take snapshots of the reaction and often requires drying of the sample prior to investigation, which can induce artefacts.

Hard X‐ray spectromicroscopy can breach the gap between these techniques. In X‐ray spectromicroscopy X‐ray absorption spectra (XAS) and X‐ray fluorescence (XRF) spectra are mapped across a sample using a focused beam. This is particularly useful in revealing local variations in the chemistry of the sample at larger length‐scales than EM‐based spectroscopy methods such as electron energy loss spectroscopy (EELS) and energy dispersive X‐ray spectroscopy (EDS).^21^, ^22^ As such, it can reveal mid‐ to long‐range chemical processes that are difficult to visualise with other techniques. This technique has already proven useful when studying battery materials ex situ,^23^, ^24^ but would offer even greater potential when capable of capturing chemical and structural dynamics in situ.

In this study, we show the application of an in situ liquid setup at the Diamond Light Source I14 hard X‐ray nanoprobe for studying dynamic chemical reactions in pure Ni oxide (LiNiO_2_) and the Ni‐rich material LiNi_x_Mn_y_Co_1‐x‐y_O_2_ (NMC811). We look at the stability of the materials under dry versus wet conditions, at room temperature and at elevated temperatures, 45°C or 80°C. The setup specifically allows us to disentangle heating effects from other factors such as cycling induced structural changes. Overall, we identify local differences in nickel speciation and lithium migration happening in the two materials. For NMC811, we observe dynamic chemical interactions inside a microfracture that differ from the expected behaviour, and a structural degradation and aging under moderate heating conditions.

## 2 MATERIALS AND METHODS

### 2.1 Sample preparation

LiNiO_2_ was prepared by calcination of Ni(OH)_2_ with 5 mol% excess LiOH. The precursors were mixed in a turbula mixer and calcined at 700°C for 6 h in a furnace under oxygen flow of 1 mL/min. Commercial NMC 811 powder was obtained from MSE Supplies used without any treatment. Commercial 1 M LiPF_6_ in EC:DMC with 2 wt% VC from Solvay was used as the electrolyte. The standard samples were made into pellets of diameter 10 mm using boron nitride.

The NMC electrodes were fabricated from slurry and coated on aluminium foil using 94 wt% NMC811 (MSE Supplies), 3 wt% polyvinylidene fluoride (PVDF) binder (Solvay 5130), and 3 wt% carbon black (Timcal C65). The electrodes have a thickness of ∼50 µm (including 15 µm Al foil), a porosity of ∼30 % and an area loading of 10 mg/cm^2^.

The samples were prepared using a Crossbeam 550 focussed ion beam scanning electron microscope (FIB‐SEM, Carl Zeiss) with a gallium ion beam. Powder samples were dusted onto a carbon tab, from which FIB lamellae were lifted out and thinned to 700 nm using a 30 kV ion beam, followed by polishing at 5 kV. Lamellae were then placed onto a MEMS chip within the FIB‐SEM chamber.

### 2.2 In situ setup

**FIGURE 1:**
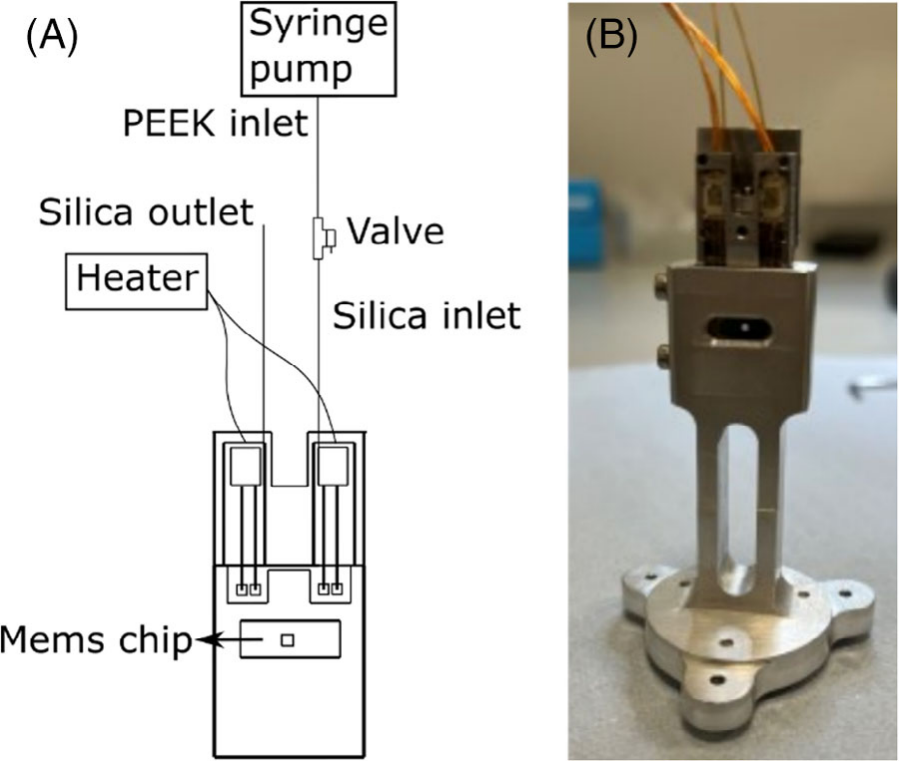
In situ liquid heater setup for use on the nanoprobe. (A) Schematic of the full setup. (B) Photo of the holder inside the mount. Setup adapted from the in situ gas system as reported in Ref. (26).

The in situ liquid setup developed at the hard X‐ray nanoprobe I14 beamline (Diamond Light Source, Didcot, UK) was configured as described previously,^25^ using an in‐house developed mount and the DENSsolutions sample holder and heating connections as previously developed (see Figure 1).^25^, ^26^ The holder was mounted on the beamline scanning stages on a kinematic mount.^27^ DENSsolutions Climate reaction nanochips (product number P.J. GH.SS2) were used, containing an 800 × 800 µm silicon nitride window of 400 nm thickness with small thinned areas of 20 nm thickness and 6 µm diameter. A platinum heating spiral of 500 µm × 500 µm was printed on the window of the bottom chip, and the chips were sealed with a viton O‐ring. Heating was done at a ramp of 5°C/min. The chips were connected to a gastight syringe (1 mL Hamilton syringe) via PEEK tubing (IDEX). The gastight syringe was used to avoid generation of HF in humid air. Electrolyte was flown into the cell at 5 µL/min using a syringe pump until the cell was filled and liquid was coming out of the outlet tube. The flow was then stopped before starting the measurements.

### 2.3 XRF and XANES mapping

All measurements were performed at the hard X‐ray nanoprobe I14 beamline (Diamond Light Source, UK).^27^ Samples were scanned by a focused X‐ray beam of 50 × 50 nm. XRF data was collected by continuous raster scanning using a four‐element silicon‐drift detector in the backscatter geometry (RaySpec, UK) with a solid collection angle of 0.8 sr. Independent XRF maps were taken at 9 keV, using a dwell time of 15 ms and a step size of 65 nm which results in approximately 1‐ to 3‐min long scans, depending on the size of the scan area. Using PyMCA software,^28^ a pixel‐by‐pixel background subtraction was performed and the fluorescence peaks were fitted to construct elemental Ni maps that reveal the morphology of the sample.

For acquisition of spatially resolved X‐ray absorption near‐edge structure (XANES) spectra a collection of 152 XRF maps were taken through the Ni K‐edge. A dwell time of 15 ms per pixel was used, with 65 nm step size, resulting in 3‐ to 4‐h long scans for the full XANES stack, depending on the size of the area imaged. Active drift compensation was used to keep the sample in the field of view during the XANES scan.^29^ Additional alignment of the resulting image stack was performed through image registration using mutual information as a metric,^30^ aligning the integrated intensity of the measured spectra from the mappings. After image alignment principal component analysis (PCA) and cluster analysis (CA) were performed on the stacked XANES maps, using the software MANTiS,^31^ to identify spectroscopically similar areas. Using MANTiS, PCA is first used to identify the eigenvectors of the spectra, that is, the principal components that together constitute the measured spectra in the image.^31^ The full spectral image can be reconstructed using a linear combination of these components, but only a subset of them is essential to do so. MANTiS can be used to determine this subset, which is then used for further analysis using CA. In CA, pixels containing a similar weighting of eigenspectra (or principal components) are clustered together in colour coded maps, where each colour represents spectrally similar pixels.^31^ The combined spectra in each cluster are then averaged and the resulting spectrum is used for further analysis. The cluster that represents the background in the image, that is, the region surrounding the FIB section where there is no sample, is identified by comparing these colour coded XANES maps with XRF maps.

The averaged spectra found through CA in MANTiS were further analysed in the programme Athena (part of the Demeter software suite^32^). First, the spectra were normalised to correct for fluctuations and variation in beam energy and intensity,^32^ which can vary across beamlines and depend on calibration of beamline optics. The offset of the edge energy *E*_0_ is determined from measuring a Ni metal foil, for which *E*_0_ is known, at the start of the experiments.^32^ All spectra are subsequently shifted by this offset. To correct for fluctuations in intensity spectra are normalised to their pre‐ and postedge absorption intensity.^33^ After normalisation, linear component fitting (LCF) is used to determine the speciation of nickel in each cluster. For this LCF procedure standards of LiNiO_2_ for Ni^3+^ and NiO for Ni^2+^ were used, both in‐house prepared as described in *Sample preparation* and measured at the I14 beamline using two gridded split ion chambers. The energy offset of all these experimentally obtained spectra was calculated against a metallic Ni foil (Goodfellow Cambridge Ltd, NI000224/3) that was measured between the ion chambers at the start of the experiments, using the first inflection point in the K_α_ absorption edge. This energy offset was then corrected for accordingly in Athena. The LCF was started using one standard, with subsequent standards being added as long as addition of more components reduced the R‐factor by 10% or more.^34^ Subsequent XANES maps for the same sample under different conditions were all taken at a slight offset with respect to each other, in addition to overview XRF scans that were taken after each XANES scan, to verify that no beam damage was observed (Figure S2). To evaluate the goodness of fit for the LCF fitting the R‐factor and reduced *χ* ^2^ were considered,^35^ as provided by Athena (Tables S1–S3). Similarly, the error in the value for the concentration of each compound was used as provided by Athena. This error was then used to create error bars indicating the uncertainty of the fit.

## 3 RESULTS AND DISCUSSION

We studied the chemical and structural stability of sections of pristine (i.e., uncycled) LiNiO_2_ and NMC811 in situ upon exposure to electrolyte and subsequent heating. We did so by mapping local changes in the oxidation state of nickel using nano‐XANES and correlating this with changes to the morphology of the sample observed through XRF images. In our analysis we focused on the ratio of Ni^3+^ to Ni^2+^, as those are the main nickel species that are expected inside this material. Here, Ni^3+^ represents LiNiO_2_, the nickel atoms when bound to lithium, and Ni^2+^ represents NiO, the nickel atoms after removal of lithium. As such, by following the nickel speciation we can infer the presence of lithium, even if the absorption K‐edge of lithium at 55 eV is too low to be detected directly using the nanoprobe.

**FIGURE 2:**
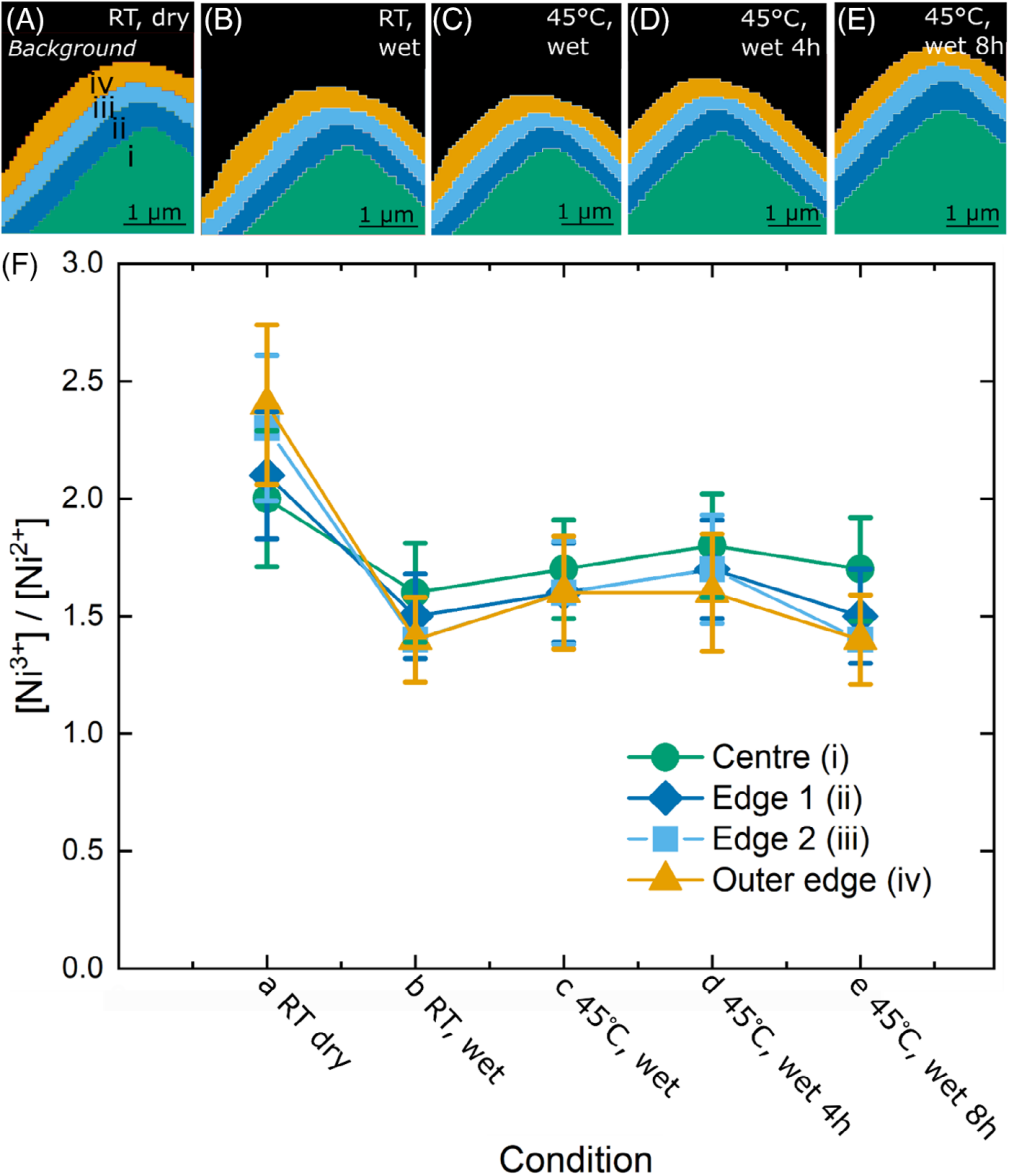
Local changes in the nickel oxidation state in a section of a LiNiO_2_ electrode upon contact with electrolyte and heating. (A–E) XANES nickel speciation maps. Areas with different nickel speciation are indicated i‐iv and by different colours. The background is indicated in black. XANES maps are taken (A) at room temperature under dry conditions, (B) at room temperature after addition of electrolyte, (C) immediately after bringing the temperature of the cell to 45°C, (D) 4 h after start of heating to 45°C and (E) 8 h after start of heating to 45°C. (F) The ratio between the amount of Ni^3+^ to Ni^2+^ for each of the areas of interest. Conditions correspond to a–e at the top of this figure and are indicated accordingly. Error bars indicate the uncertainty of the fit as provided by Athena.

From the fitting parameters in the LCF fit (Tables S1–S3), we confirm that Ni^3+^ and Ni^2+^ are the main nickel components found in these samples. However, some deviations of the fit with respect to the measured spectra can be found (Figure S3), indicating that there is at least one more minor compound in this sample. This gives rise to an error in the fit, which is indicated by the error bars in Figure 2F.

By mapping the XANES signal across the nickel absorption edge, we track changes in the oxidation state of the nickel atoms. Through principal component analysis and subsequent cluster analysis of the XANES spectra, we identify areas with different nickel chemistry. For the LiNiO_2_ sample, we find four relevant areas (Figure 2A–E): the bulk of the sample in the centre (i), and three layers on the edge with an inner edge closest to the bulk (ii), a middle edge on top of that (iii) and an outer edge that forms the surface of the section which is directly exposed to the electrolyte (iv).

**FIGURE 3:**
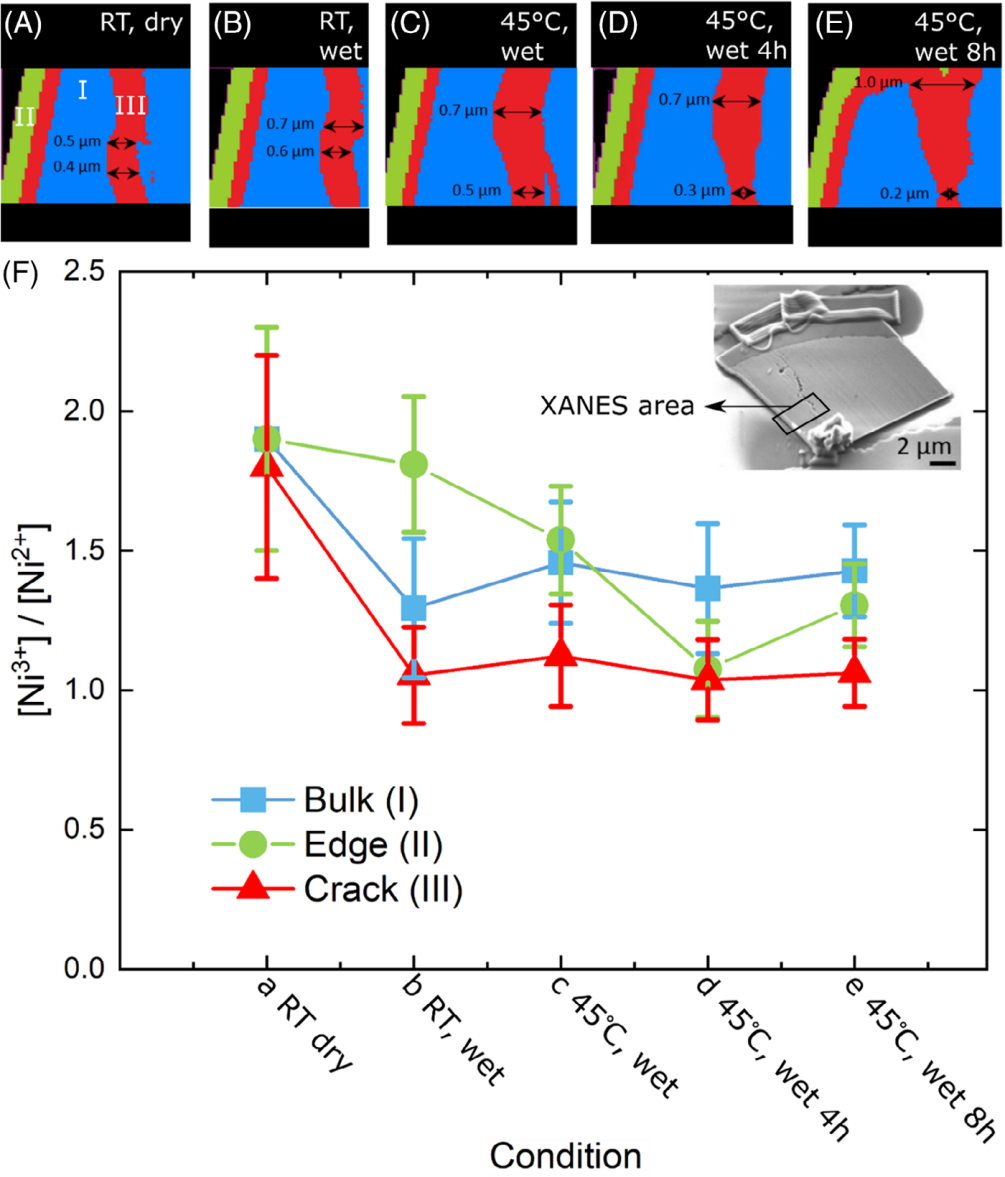
Local changes in the nickel oxidation state in a cracked section of an NMC811 electrode upon contact with electrolyte and heating. (a–e) XANES nickel speciation maps. Regions of interest in the XANES maps are indicated: I inner bulk of the material, II outer edge and III cracked area. Maximum and minimum width of the XANES cluster corresponding to the cracked region is indicated in each map. The background is indicated in black. XANES maps are taken (A) at room temperature under dry conditions, (B) at room temperature after addition of electrolyte, (C) immediately after bringing the temperature of the cell to 45°C, (D) 4 h after start of heating to 45°C and (E) 8 h after start of heating to 45°C. (F) The ratio between the amount of Ni^3+^ to Ni^2+^ for each of the areas of interest. Conditions correspond to a–e at the top of this figure and are indicated accordingly. Error bars indicate the uncertainty of the fit as provided by Athena.

We also study NMC811 samples that have been doped with manganese and cobalt (LiNi_0.8_Mn_0.1_Co_0.1_O_2_) to provide a more stable material compared to pure LiNiO_2_. Additionally, one of the NMC811 samples measured here contains a preexisting fracture that shows how different the electrolyte interaction is for the sample surface versus a fractured area (Figure **3**). For this sample, XANES maps reveal three regions with different chemical build‐up (Figure 3A–E): (I) the inner bulk of the sample, (II) an outer edge, and (III) the fractured area which has identical Ni speciation to a thin region sitting between the outer edge and the bulk.

Overall, the fitted XANES spectra reveal a marked loss in Ni^3+^ for both LiNiO_2_ and NMC811 (Figures 2F and 3F), and thus in lithium content, when electrolyte is first introduced to the system. This marks a process of lithium migration and the formation of an SEI layer on the material. However, the samples show a different behaviour of edge versus bulk. An initial difference in Ni^3+^ content between edge and bulk can be expected as LiNiO_2_ is an air sensitive material and is likely to form oxidised forms upon air exposure, particularly on the outer edges. Upon introduction of the electrolyte LiNiO_2_ loses more Ni^3+^ at the edge that is directly exposed to the electrolyte, compared to the inner bulk of the material (Figure 2F). In contrast, the cracked NMC811 section initially barely shows any drop in Ni^3+^ of the edge at all but a strong conversion of Ni^3+^ to Ni^2+^ in the bulk and fractured area (Figure 3F). Over time, the edge of NMC811 then follows the trend of the rest of the sample and lowers in lithium content too (Figure 3F). This difference between the two materials is most pronounced when comparing LiNiO_2_ to the cracked NMC811 sample, where the Ni^3+^ content at the edge initially barely changes, but a similar trend is observed for an NMC811 section without fracture (Figure S1). There are various possible explanations for this observation. The difference between LiNiO_2_ and NMC811 could be an indication of dissolution of transition metals (Ni, Co, Mn), a well‐known process in Ni‐rich cathode materials that is normally associated with aging of the material and can severely damage the capacity of the battery during cycling when the metals deposit on the anode.^15^, ^37^ Alternatively, it is possible that the difference stems from movement of Ni^2+^ ions that block channels for lithium diffusion or migration, causing variation in lithium migration pathways between the two materials.^16^

**FIGURE 4:**
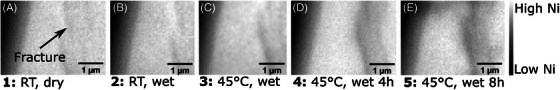
Structural degradation of NMC811 at 45°C. XRF maps showing the X‐ray fluorescence intensity at the Ni‐K edge in each condition for a FIB section of an NMC811 sample with a fracture, measured under different conditions. High fluorescence, indicating high concentration of nickel, is indicated by bright pixels. The section imaged (A) at room temperature, in dry condition, (B) at room temperature after addition of the electrolyte, (C) immediately after bringing the temperature of the cell to 45°C, (D) 4 h after start of heating to 45°C and (E) 8 h after start of heating to 45°C.

In addition to the chemistry of the edge versus bulk, we also observe interesting dynamics in the fracture of the NMC811 sample (Figures 3 and 4). Microfractures are a key problem for Ni‐rich cathode materials, as they appear upon cycling of the material due to volume expansion. It is typically assumed that such microfractures contribute to capacity fading as they expose the unprotected inside of the cathode to electrolyte. In our case the crack was formed during the FIB preparation. Nevertheless, the principle of the unprotected inside of the material still applies. It might be expected that for this uncycled sample the fracture acts as a second edge area. However, this is clearly not the case here. Instead, the fracture area shows a strong decrease in Ni^3+^ (Figure 3F), which is not observed for the edge area (Figure 3F). The speciation map of the cracked also changes shape (Figures 3A–E and 4). Overall, it seems that microfracture behaviour for Ni‐rich cathodes is more complicated than a simple mechanism of unprotected electrolyte exposure.

**FIGURE 5:**
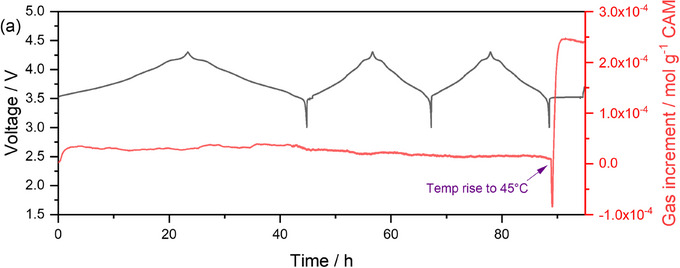
Operando analysis of gas evolution during formation cycles and subsequent gas evolution during storage at 45°C using PAT‐Cell‐Press EL‐Cell®.

Interestingly, the morphology of the fractured NMC811 sample does change upon heating, although it does not directly mirror the changes in chemical speciation. We observe a gradual loss of nickel around the fracture in the NMC811 sample, as observed through the XRF elemental maps of the Ni‐K edge (Figure 4A–E). Additionally, there is a loss of nickel observed in a horizontal area following the top of Figure 4E. The latter might be considered an indication of beam damage effects, as this horizontal region follows the scanning direction, but here beam damage effects would typically be expected to appear more strongly at the bottom right of the image, where the beam rests between scans. This is not observed here. To verify that this degradation of the sample at 45°C occurs without the X‐ray beam (i.e., to check for beam effects), we follow the evolution of gas inside the cell during cycling (Figure 5). Gas evolution typically occurs as a result of degradation and is normally observed during hot storage tests.^10^ Here, we find a sharp increase in the amount of gas evolved when the cell was taken at 45°C at open circuit voltage following the initial conditioning cycles (1 × C/20 + 2 × C/10). This excludes explanations based fully on beam damage effects, and thus further confirms the results from the XRF maps that indicate that these cathodes exhibit accelerated aging at 45°C.

**FIGURE 6:**
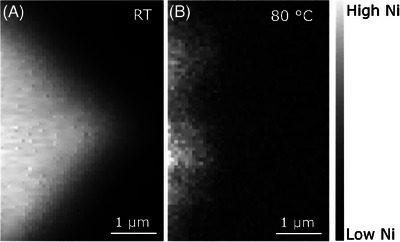
Structural degradation of NMC811 at 80°C. XRF maps showing the X‐ray fluorescence intensity at the Ni‐K edge in each condition for a FIB section of an NMC811 sample, measured under different conditions. High fluorescence, indicating high concentration of nickel, is indicated by bright pixels. The section imaged (A) at room temperature in electrolyte and (B) at 80°C in electrolyte.

Next, we study a section of NMC811 without fracture, and heat it to 80°C to accelerate aging further and probe changes in the chemical composition at the cathode–electrolyte interface. We then use XRF maps of the Ni‐K edge to capture the onset of the degradation process. Directly upon reaching this temperature, the XRF map shows an almost complete degradation of the sample (Figure 6A and B). The degradation occurs at a timescale that only allows for an analysis of the elemental distribution and morphology of the sample, but not the chemical speciation of Ni.

Normally, the onset of temperature induced degradation of Ni‐rich batteries is expected around 60°C–90°C.^38^, ^39^, ^40^ This process then starts with degradation of the passivation layer^38^, ^40^ followed by the anode,^39^ with cathode degradation reported between 140–300°C.^39^, ^41^ Strikingly, our XRF image at 80°C for the pristine NMC811 cathodes studied here shows complete destruction of the sample. This occurs at lower temperatures than expected, which is likely due to the larger portion of the sample involved in forming the passivation layer because of interaction with the electrolyte that starts degrading at these temperatures.

## 4 CONCLUSIONS

We demonstrate the use of an in situ setup for liquid imaging using a hard X‐ray nanoprobe, to visualise the electrode–electrolyte interface formation and thermally induced chemical degradation of Ni‐rich cathode materials. Through XANES mapping of the Ni‐K edge under dry versus wet conditions, at room temperature, 45°C or 80°C, we observe dynamic changes in the ratio of Ni^3+^ versus Ni^2+^ for bulk versus surface area. We reveal a different interaction between electrolyte and LiNiO_2_ than for electrolyte and NMC811. Notably, LiNiO_2_ shows more Ni^3+^ loss from the surface while NMC811 shows more Ni^3+^ loss from the bulk or fractured area. A preexisting fracture in an NMC811 section does not exhibit the chemical patterns associated with the surface of the material, contrary to what might be expected. Lastly, heating of the material to 45°C or 80°C induces structural degradation and gassing of the sample that can be observed through XRF mapping and cycling tests. Overall, the DENSsolutions in situ liquid cell shows distinct changes are happening at the electrolyte–NMC interface compared to bulk that allows tailoring of particle coating and bulk according to the structural changes observed in situ within the electrolyte medium to improve their stability during long‐term operation.

## Supporting information



Supporting Information

## Data Availability

The data that support the findings of this study are available from the corresponding author upon reasonable request.

## References

[1] Zhao, T. , Jiang, Y. , Luo, S. , Ying, Y. , Zhang, Q. , Tang, S. , Chen, L. , Xia, J. , Xue, P. , Zhang, J.‐J. , Sun, S.‐G. , & Liao, H.‐G. (2023). On‐chip gas reaction nanolab for *in situ* TEM observation. Lab on A Chip, 23, 3768–3777. 37489871 10.1039/d3lc00184a

[2] Van Omme, J. T. , Wu, H. , Sun, H. , Beker, A. F. , Lemang, M. , Spruit, R. G. , Maddala, S. P. , Rakowski, A. , Friedrich, H. , Patterson, J. P. , & Pérez Garza, H. H. (2020). Liquid phase transmission electron microscopy with flow and temperature control. Journal of Materials Chemistry C. Materials, 8, 10781–10790.

[3] Daigaku, T. (2016). IEEE nanotechnology council & institute of electrical and electronics engineers. In: *Proceedings of the* 11th Annual International Conference on Nano/Micro Engineered and Molecular Systems (NEMS) . IEEE.

[4] Yang, Y. , Louisia, S. , Yu, S. , Jin, J. , Roh, I. , Chen, C. , Fonseca Guzman, M. V. , Feijóo, J. , Chen, P.‐C. , Wang, H. , Pollock, C. J. , Huang, X. , Shao, Y.‐T. , Wang, C. , Muller, D. A. , Abruña, H. D. , & Yang, P. (2023). Operando studies reveal active Cu nanograins for CO2 electroreduction. Nature, 614, 262–269. 36755171 10.1038/s41586-022-05540-0

[5] Shan, H. , Gao, W. , Xiong, Y. , Shi, F. , Yan, Y. , Ma, Y. , Shang, W. , Tao, P. , Song, C. , Deng, T. , Zhang, H. , Yang, D. , Pan, X. , & Wu, J. (2018). Nanoscale kinetics of asymmetrical corrosion in core‐shell nanoparticles. Nature Communications, 9, 1011. 10.1038/s41467-018-03372-z PMC5843659 29520056

[6] Divitini, G. , Cacovich, S. , Matteocci, F. , Cinà, L. , Di Carlo, A. , & Ducati, C. (2016). In situ observation of heat‐induced degradation of perovskite solar cells. Nature Energy, 1, 15012. 10.1038/NENERGY.2015.12

[7] Mehdi, B. L. , Qian, J. , Nasybulin, E. , Park, C. , Welch, D. A. , Faller, R. , Mehta, H. , Henderson, W. A. , Xu, W. , Wang, C. M. , Evans, J. E. , Liu, J. , Zhang, J.‐G. , Mueller, K. T. , & Browning, N. D. (2015). Observation and quantification of nanoscale processes in lithium batteries by operando electrochemical (S)TEM. Nano Letters, 15, 2168–2173. 25705928 10.1021/acs.nanolett.5b00175

[8] Yuan, Y. , Amine, K. , Lu, J. , & Shahbazian‐Yassar, R. (2017). Understanding materials challenges for rechargeable ion batteries with in situ transmission electron microscopy. Nature Communications, 8, 15806.

[9] Jiang, M. , Danilov, D. L. , Eichel, R. A. , & Notten, P. H. L. (2021). A review of degradation mechanisms and recent achievements for Ni‐rich cathode‐based Li‐ion batteries. Advanced Energy Materials, 11, 2103005. 10.1002/aenm.202103005

[10] Rowden, B. , & Garcia‐Araez, N. (2020). A review of gas evolution in lithium ion batteries. Energy Reports, 6, 10–18.

[11] Lee, R.‐C. , Franklin, J. , Tian, C. , Nordlund, D. , Doeff, M. , & Kostecki, R. (2021). The origin of impedance rise in Ni‐rich positive electrodes for lithium‐ion batteries. Journal of Power Sources, 498, 229885.

[12] Heenan, T. M. M. , Wade, A. , Tan, C. , Parker, J. E. , Matras, D. , Leach, A. S. , Robinson, J. B. , Llewellyn, A. , Dimitrijevic, A. , Jervis, R. , Quinn, P. D. , Brett, D. J. L. , & Shearing, P. R. (2020). Identifying the origins of microstructural defects such as cracking within Ni‐rich NMC811 cathode particles for lithium‐ion batteries. Advanced Energy Materials, 10, 10.1002/aenm.202002655

[13] Bianchini, M. , Fauth, F. , Hartmann, P. , Brezesinski, T. , & Janek, J. (2020). An *in situ* structural study on the synthesis and decomposition of LiNiO_2_ . Journal of Materials Chemistry A. Materials, 8, 1808–1820.

[14] Kim, T. , Ono, L. K. , Fleck, N. , Raga, S. R. , & Qi, Y. (2018). Transition metal speciation as a degradation mechanism with the formation of a solid‐electrolyte interphase (SEI) in Ni‐rich transition metal oxide cathodes. Journal of Materials Chemistry A. Materials, 6, 14449–14463.

[15] Jung, R. , Linsenmann, F. , Thomas, R. , Wandt, J. , Solchenbach, S. , Maglia, F. , Stinner, C. , Tromp, M. , & Gasteiger, H. A. (2019). Nickel, manganese, and cobalt dissolution from Ni‐rich NMC and their effects on NMC622‐graphite cells. Journal of the Electrochemical Society, 166, A378–A389.

[16] Manthiram, A. , Knight, J. C. , Myung, S. , Oh, S. , & Sun, Y. (2016). Nickel‐rich and lithium‐rich layered oxide cathodes: Progress and perspectives. Advanced Energy Materials, 6, 1501010.

[17] Li, S. , Yao, Z. , Zheng, J. , Fu, M. , Cen, J. , Hwang, S. , Jin, H. , Orlov, A. , Gu, L. , Wang, S. , Chen, Z. , & Su, D. (2020). Direct observation of defect‐aided structural evolution in a nickel‐rich layered cathode. Angewandte Chemie‐International Edition, 59, 22092–22099. 32743947 10.1002/anie.202008144

[18] Kim, U.‐H. , Ryu, H.‐H. , Kim, J.‐H. , Mücke, R. , Kaghazchi, P. , Yoon, C. S. , & Sun, Y.‐K. (2019). Microstructure‐controlled Ni‐rich cathode material by microscale compositional partition for next‐generation electric vehicles. Advanced Energy Materials, 9, 1803902.

[19] de Jonge, N. , Houben, L. , Dunin‐Borkowski, R. E. , & Ross, F. M. (2018). Resolution and aberration correction in liquid cell transmission electron microscopy. Nature Reviews Materials, 4, 61–78.

[20] Holtz, M. E. , Yu, Y. , Gao, J. , Abruña, H. D. , & Muller, D. A. (2013). *In situ* electron energy‐loss spectroscopy in liquids. Microscopy and Microanalysis, 19, 1027–1035. 23721691 10.1017/S1431927613001505

[21] Pattammattel, A. , Tappero, R. , Ge, M. , Chu, Y. S. , Huang, X. , Gao, Y. , & Yan, H. (2020). High‐sensitivity nanoscale chemical imaging with hard X‐ray nano‐XANES. Science Advances, 6, eabb3615. 32917679 10.1126/sciadv.abb3615 PMC11206466

[22] Lin, F. , Liu, Y. , Yu, X. , Cheng, L. , Singer, A. , Shpyrko, O. G. , Xin, H. L. , Tamura, N. , Tian, C. , Weng, T.‐C. , Yang, X.‐Q. , Meng, Y. S. , Nordlund, D. , Yang, W. , & Doeff, M. M. (2017). Synchrotron X‐ray analytical techniques for studying materials electrochemistry in rechargeable batteries. Chemical Reviews, 117, 13123–13186. 28960962 10.1021/acs.chemrev.7b00007

[23] Liu, X. , Wang, D. , Liu, G. , Srinivasan, V. , Liu, Z. , Hussain, Z. , & Yang, W. (2013). Distinct charge dynamics in battery electrodes revealed by in situ and operando soft X‐ray spectroscopy. Nature Communications, 4, 2568. 10.1038/ncomms3568 PMC3806410 24100759

[24] Shearing, P. , Wu, Y. , Harris, S. J. , & Brandon, N. (2011). In situ X‐ray spectroscopy and imaging of battery materials. Interface Magazine, 20, 43–47.

[25] Van De Kerkhof, G. T. , Walker, J. M. , Agrawal, S. , Clarke, S. M. , Sk, M. H. , Craske, D. J. , Lindsay, R. , Dowhyj, M. , Osundare, A. , Schuster, M. E. , & Parker, J. E. (2023). An *in situ* liquid environment for synchrotron hard X‐ray nanoprobe microscopy. Materials at High Temperatures, 40, 371–375.

[26] Parker, J. E. , Gomez‐Gonzalez, M. , Van Lishout, Y. , Islam, H. , Duran Martin, D. , Ozkaya, D. , Quinn, P. D. , & Schuster, M. E. (2022). A cell design for correlative hard X‐ray nanoprobe and electron microscopy studies of catalysts under *in situ* conditions. Journal of Synchrotron Radiation, 29, 431–438. 35254306 10.1107/S1600577521013576 PMC8900865

[27] Quinn, P. D. , Alianelli, L. , Gomez‐Gonzalez, M. , Mahoney, D. , Cacho‐Nerin, F. , Peach, A. , & Parker, J. E. (2021). The hard X‐ray nanoprobe beamline at diamond light source. Journal of Synchrotron Radiation, 28, 1006–1013. 33950009 10.1107/S1600577521002502 PMC8127369

[28] Solé, V. A. , Papillon, E. , Cotte, M. , Walter, P. , & Susini, J. (2007). A multiplatform code for the analysis of energy‐dispersive X‐ray fluorescence spectra. Spectrochim Acta Part B At Spectrosc, 62, 63–68.

[29] Quinn, P. D. , Gomez‐Gonzalez, M. , Cacho‐Nerin, F. , & Parker, J. E. (2021). Beam and sample movement compensation for robust spectro‐microscopy measurements on a hard X‐ray nanoprobe. Journal of Synchrotron Radiation, 28, 1528–1534. 34475300 10.1107/S1600577521007736 PMC8415335

[30] Viola, P. , & Wells, III, W. M. (1997). Alignment by maximization of mutual information. International Journal of Computer Vision, 9, 137–154.

[31] Lerotic, M. , Mak, R. , Wirick, S. , Meirer, F. , & Jacobsen, C. (2014). *MANTiS* : A program for the analysis of X‐ray spectromicroscopy data. Journal of Synchrotron Radiation, 21, 1206–1212. 25178014 10.1107/S1600577514013964

[32] Ravel, B. , & Newville, M. (2005). *ATHENA*, *ARTEMIS*, *HEPHAESTUS* : Data analysis for X‐ray absorption spectroscopy using *IFEFFIT* . Journal of Synchrotron Radiation, 12, 537–541. 15968136 10.1107/S0909049505012719

[33] Newville, M. (2004). *Fundamentals of XAFS*. Consortium for Advanced Radiation Sources. Chicago, IL: University of Chicago.

[34] Gomez‐Gonzalez, M. A. , Da Silva‐Ferreira, T. , Clark, N. , Clough, R. , Quinn, P. D. , & Parker, J. E. (2023). Toward understanding the environmental risks of combined microplastics/nanomaterials exposures: Unveiling ZnO transformations after adsorption onto polystyrene microplastics in environmental solutions. Global Challenges, 7, 2300036. 37635705 10.1002/gch2.202300036 PMC10448137

[35] Kelly, S. D. , & Ravel, B. (2008). Analysis of soils and minerals using X‐ray absorption spectroscopy. In A. L. Ulery & L. R. Drees (Eds.), Methods of soil analysis. Part 5. Mineralogical methods. SSSA Book Series. Soil Science Society of America, Inc.

[36] Wu, Z. , Zhang, C. , Yuan, F. , Lyu, M. , Yang, P. , Zhang, L. , Zhou, M. , Wang, L. , Zhang, S. , & Wang, L. (2024). Ni‐rich cathode materials for stable high‐energy lithium‐ion batteries. Nano Energy, 126, 109620.

[37] Pieczonka, N. P. W. , Liu, Z. , Lu, P. , Olson, K. L. , Moote, J. , Powell, B. R. , & Kim, J.‐H. (2013). Understanding transition‐metal dissolution behavior in LiNi 0.5Mn1.5O4 high‐voltage spinel for lithium ion batteries. Journal of Physical Chemistry C, 117, 15947–15957.

[38] Feng, X. , Fang, M. , He, X. , Ouyang, M. , Lu, L. , Wang, H. , & Zhang, M. (2014). Thermal runaway features of large format prismatic lithium ion battery using extended volume accelerating rate calorimetry. Journal of Power Sources, 255, 294–301.

[39] Wang, Q. , Jiang, L. , Yu, Y. , & Sun, J. (2019). Progress of enhancing the safety of lithium ion battery from the electrolyte aspect. Nano Energy, 55, 93–114.

[40] Genieser, R. , Ferrari, S. , Loveridge, M. , Beattie, S. D. , Beanland, R. , Amari, H. , West, G. , & Bhagat, R. (2018). Lithium ion batteries (NMC/graphite) cycling at 80°C: Different electrolytes and related degradation mechanism. Journal of Power Sources, 373, 172–183.

[41] Jhu, C. Y. , Wang, Y. W. , Wen, C. Y. , & Shu, C. M. (2012). Thermal runaway potential of LiCoO_2_ and Li(Ni_1/3_Co_1/3_Mn_1/3_)O_2_ batteries determined with adiabatic calorimetry methodology. Applied Energy, 100, 127–131.

