# Supplementary material for: Hard X‐ray spectromicroscopy of Ni‐rich cathodes under in situ liquid heating conditions
Source: J Microsc. 2025 Mar 26;299(1):16–24. doi: 10.1111/jmi.13403 (PMC12166338; doi:10.1111/jmi.13403)
Supplement: Supplementary file 1 — Supporting Information [file JMI-299-16-s001.docx]

**Supplementary information**


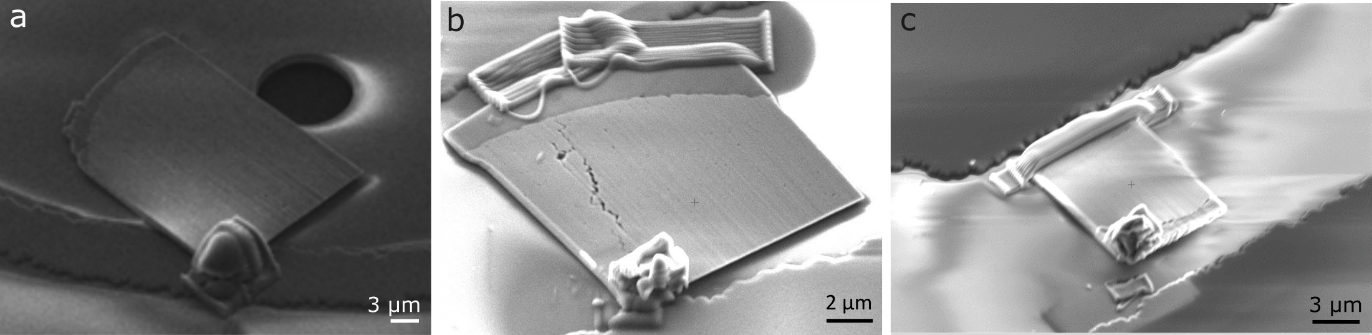
**Figure S1: SEM images of the FIB sections measured in this study**. All sections as mounted onto the *in situ* chips. a) NMC811 without fracture, b) NMC811 with fracture and c) pure LiNiO2.


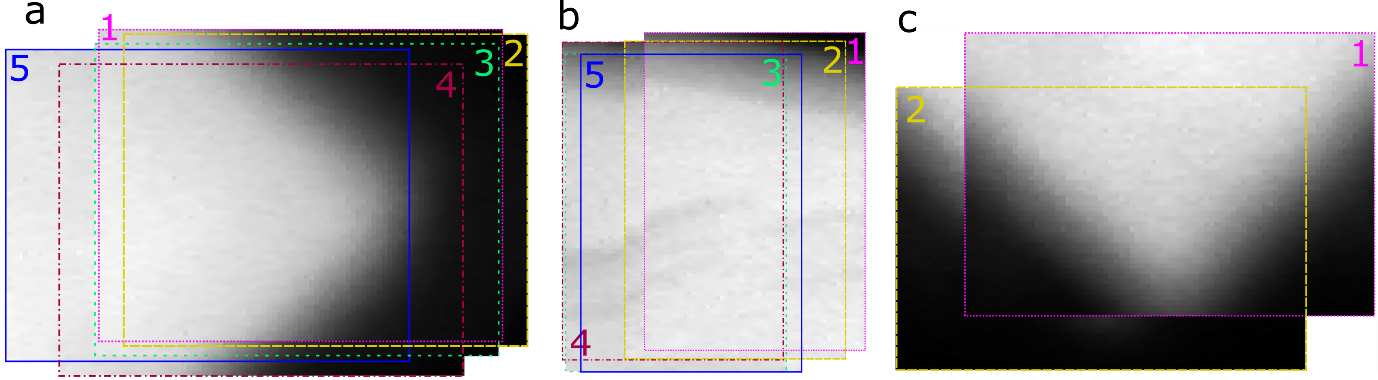


**Figure S2: XRF images showing the region of XANES mapping for each sample and each experimental condition.** Images taken halfway during the measurement, at the absorption edge. Samples are a) LiNiO2, b) NMC811 with a pre-existing crack and c) NMC811 without a crack. Conditions 1-5 correspond to (1) room temperature dry, (2) room temperature wet, (3) 45℃ wet at t=0, (4) 45℃ wet at t=4h and (5) 45℃ wet at t=8h.

**Table 1: Fitting parameters of the LiNiO_2_ sample.** R-factor and reduced chi squared for the LCF performed in Athena, corresponding the data in Figure 2f (main text).

|  |  | **Centre (i)** | **Edge 1 (ii)** | **Edge 2 (iii)** | **Outer edge (iv)** |
| --- | --- | --- | --- | --- | --- |
| **Cond. 1** | *R-factor* | 0.004055 | 0.0035789 | 0.0034247 | 0.0035945 |
|  | *Red. chi-squared* | 0.0009749 | 0.000854 | 0.0008209 | 0.0008571 |
| **Cond. 2** | *R-factor* | 0.0037862 | 0.0037664 | 0.0042761 | 0.0046962 |
|  | *Red. chi-squared* | 0.0009295 | 0.0009485 | 0.0010827 | 0.0011907 |
| **Cond. 3** | *R-factor* | 0.0037449 | 0.0041095 | 0.0048322 | 0.0054083 |
|  | *Red. chi-squared* | 0.0009154 | 0.0010126 | 0.0011998 | 0.00135 |
| **Cond. 4** | *R-factor* | 0.0034877 | 0.0039126 | 0.0047651 | 0.005897 |
|  | *Red. chi-squared* | 0.0008336 | 0.0009464 | 0.0011657 | 0.0014989 |
| **Cond. 5** | *R-factor* | 0.0039416 | 0.0043087 | 0.0046848 | 0.0048338 |
|  | *Red. chi-squared* | 0.0009715 | 0.0010704 | 0.0011652 | 0.0011957 |

**Table 2: Fitting parameters of the cracked NMC811 sample.** R-factor and reduced chi squared for the LCF performed in Athena, corresponding the data in Figure 3f (main text).

|  |  | **Bulk (I)** | **Crack (II)** | **Edge (III)** |
| --- | --- | --- | --- | --- |
| **Cond. 1** | *R-factor* | 0.007345 | 0.0095255 | 0.0086794 |
|  | *Red. chi-squared* | 0.001775 | 0.0023062 | 0.0020867 |
| **Cond. 2** | *R-factor* | 0.007726 | 0.0044126 | 0.003896 |
|  | *Red. chi-squared* | 0.001859 | 0.0009576 | 0.0009251 |
| **Cond. 3** | *R-factor* | 0.005644 | 0.0027317 | 0.0044363 |
|  | *Red. chi-squared* | 0.001354 | 0.0006206 | 0.0010557 |
| **Cond. 4** | *R-factor* | 0.006165 | 0.0054969 | 0.0035626 |
|  | *Red. chi-squared* | 0.001471 | 0.0012935 | 0.0008302 |
| **Cond. 5** | *R-factor* | 0.003717 | 0.0030979 | 0.0034625 |
|  | *Red. chi-squared* | 0.000854 | 0.0006964 | 0.0008004 |

**Table 3: Fitting parameters of the intact NMC811 sample.** R-factor and reduced chi squared for the LCF performed in Athena, corresponding the data in Figure S4.

|  |  | **Centre (i)** | **Edge 1 (ii)** | **Edge 2 (iii)** | **Outer edge (iv)** |
| --- | --- | --- | --- | --- | --- |
| **Cond. 1** | *R-factor* | 0.003832 | 0.0049335 | 0.0063701 | 0.006708 |
|  | *Red. chi-squared* | 0.001021 | 0.0013504 | 0.0017885 | 0.0018752 |
| **Cond. 2** | *R-factor* | 0.032938 | 0.027911 | 0.022615 | 0.020062 |
|  | *Red. chi-squared* | 0.010034 | 0.0083108 | 0.0065542 | 0.0057421 |


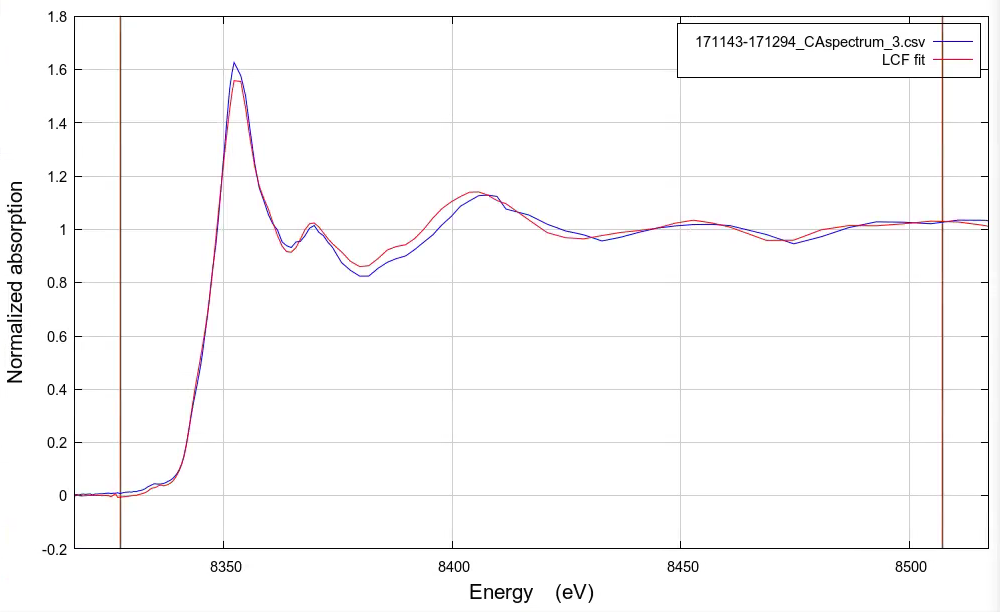


**Figure S3: Example of the quality of the LCF fit.** Normalised absorption spectrum for the edge area (III) of the cracked NMC811 section. The blue line shows the spectrum for this region, the red line shows the fit using the Ni^3+^ and Ni^2+^ standards.


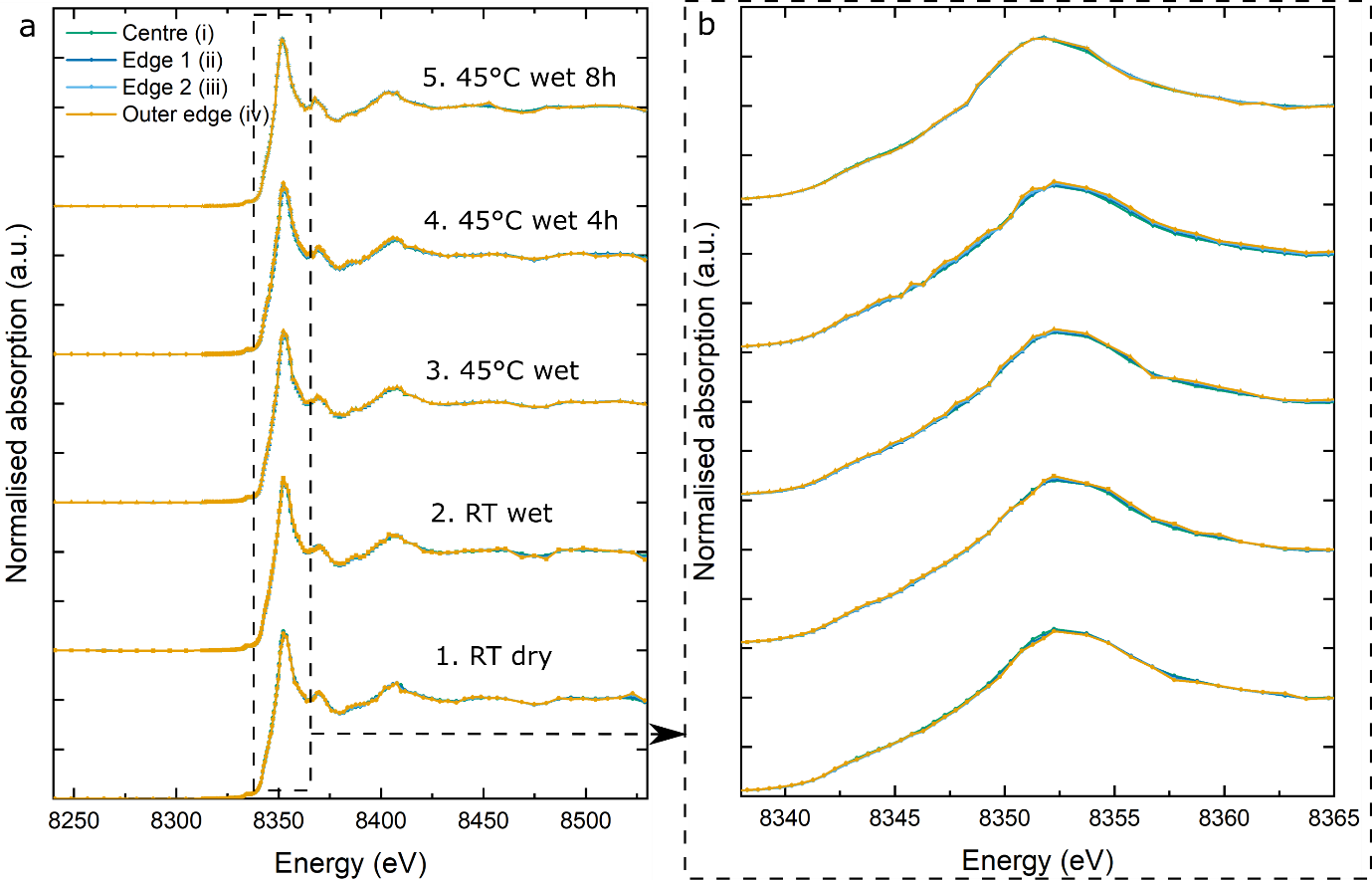


**Figure S4: XANES spectra corresponding to the LiNiO_2_ sample.** a) Full spectra for each condition and each area. b) XANES spectra zoomed in on the absorption edge to better distinguish small differences.


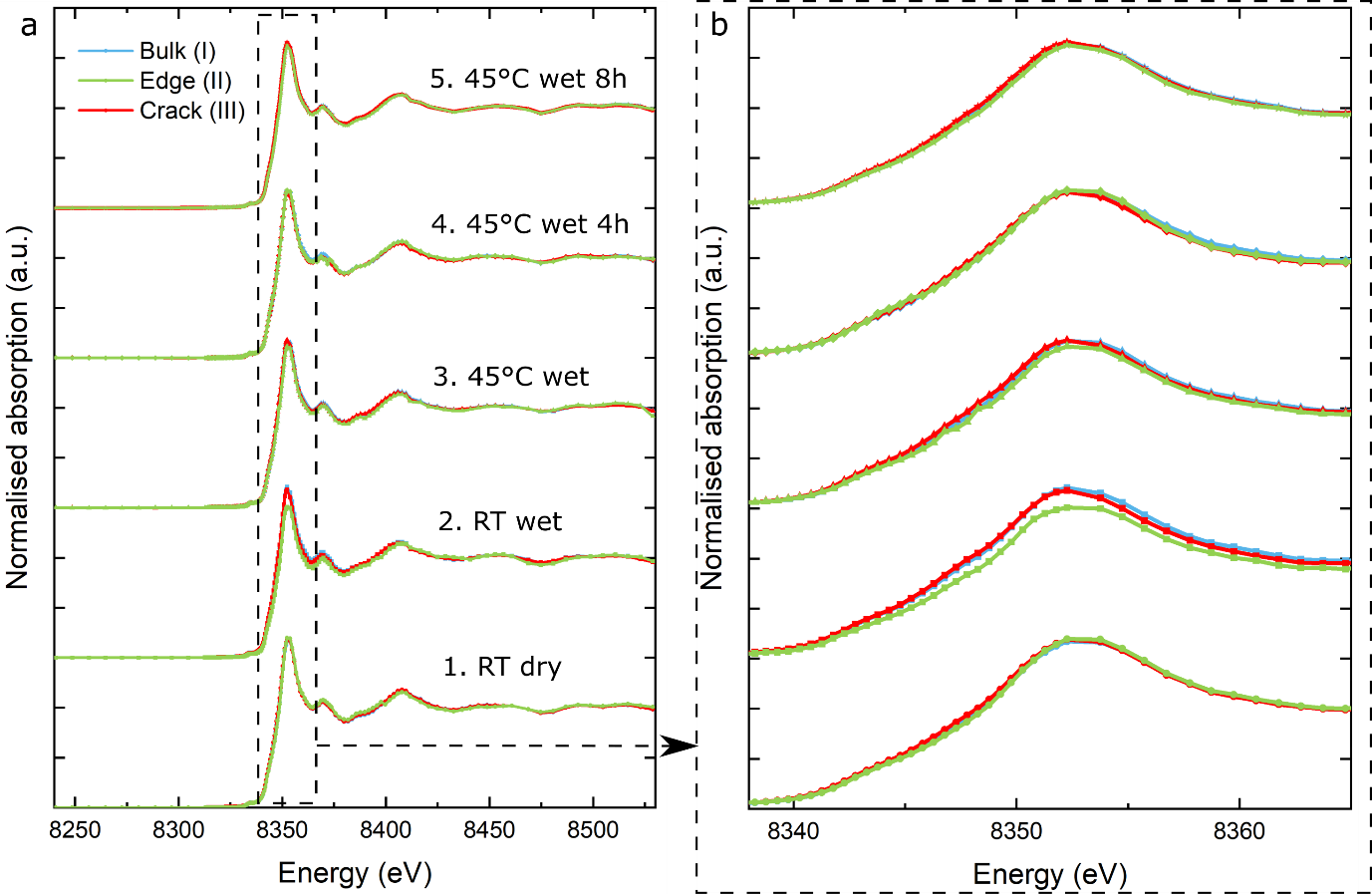


**Figure S5:** **XANES spectra corresponding to the fractured NMC811 sample.** Full spectra for each condition and each area. b) XANES spectra zoomed in on the absorption edge to better distinguish small differences.


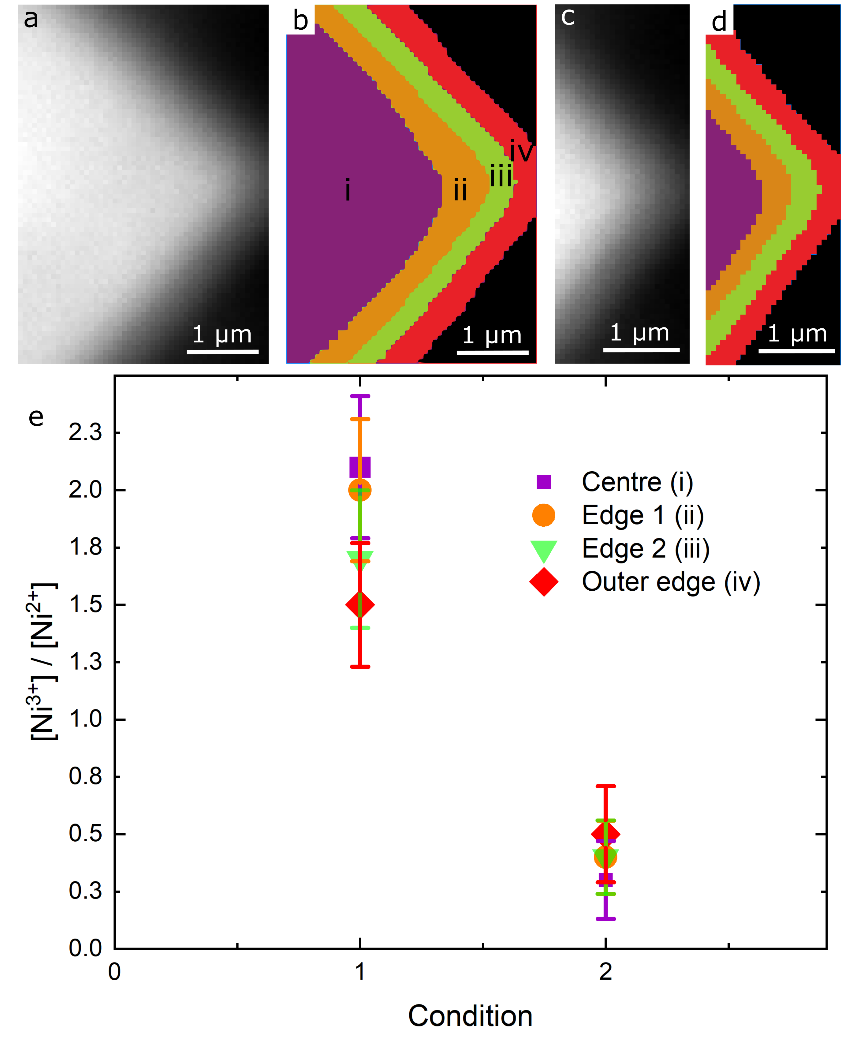


**Figure S6: Local changes in the nickel oxidation state in a cracked section of an NMC811 electrode, dry and upon contact with electrolyte.** a) and b) show the sample under dry conditions, at room temperature, and c) and d) at room temperature but exposed to electrolyte. a) and c) show XRF maps of the Ni k edge, b) and d) show the nickel XANES speciation maps, with each colour a different cluster. e) shows the ratio between the amount of Ni^3+^ to Ni^2+^ for each of the areas of interest. Error bars indicate the uncertainty of the fit.


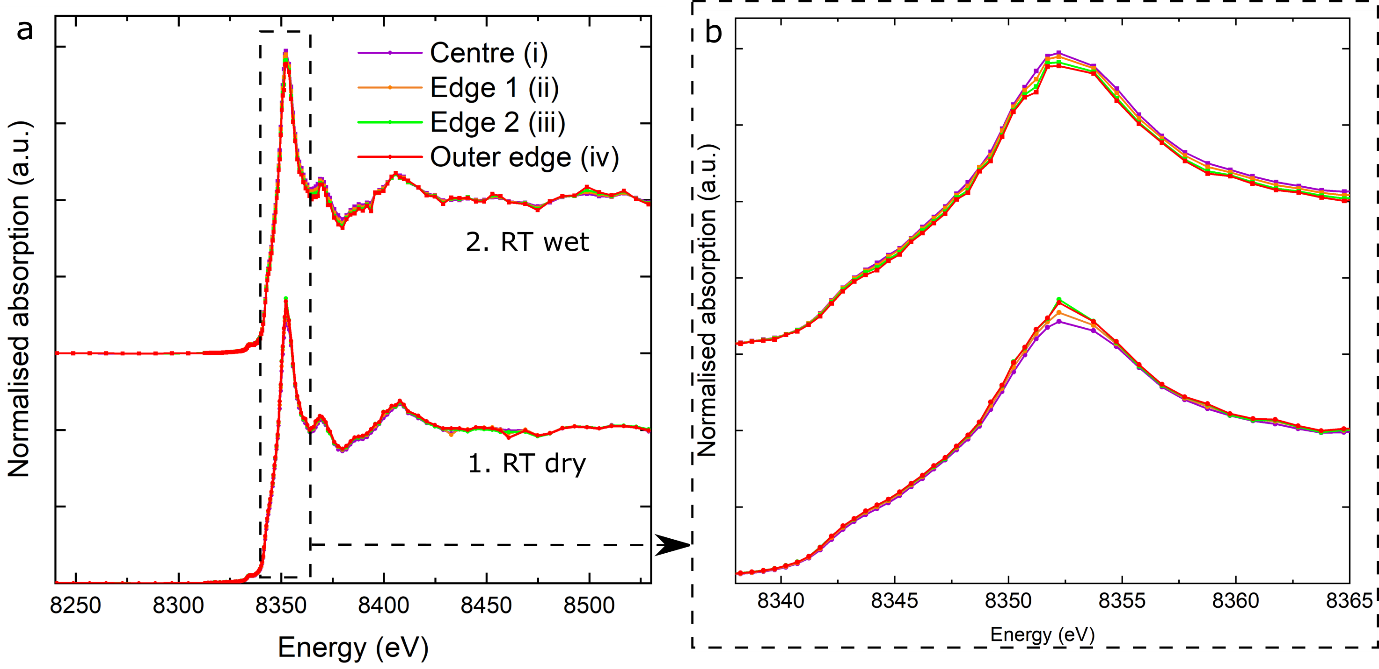


**Figure S7:** **XANES spectra corresponding to the unfractured NMC811 sample.** Full spectra for each condition and each area. b) XANES spectra zoomed in on the absorption edge to better distinguish small differences.
